# Differences in Eating and Body Image Disturbances and Sociocultural Stressors in Sexual Minoritized Men by Sexual Self-Labels Submit to *Discover Mental Health*

**DOI:** 10.21203/rs.3.rs-9216955/v1

**Published:** 2026-04-17

**Authors:** Kathryn Pasquariello, Vivian A. Lee, Benjamin F. Shepherd, Alexa Summersill, Reza N. Sahlan, Nina Michon, Urvashi Dixit, Rachel R. Henderson, Tracy Tylka, Jinbo He, Wesley R. Barnhart

**Affiliations:** 1 Department of Psychology, Suffolk University, Boston, MA, USA; 2 Department of Medical Education, Graduate School of Biomedical Sciences, Lake Erie College of Osteopathic Medicine, Erie, PA, USA; 3 Department of Social and Behavioral Sciences, Yale School of Public Health, New Haven, CT, USA; 4 Department of Psychology, Middle Tennessee State University, Murfreesboro, TN, USA; 5 Department of Counseling, School, and Educational Psychology, Graduate School of Education, University at Buffalo-SUNY, Buffalo, NY, USA; 6 Department of Counseling Psychology, Wayne State University, Detroit, MI, USA; 7 Department of Psychology, University of South Alabama, Mobile, AL, USA; 8 Department of Psychology, The Ohio State University, Columbus, OH, USA; 9 Department of Biosciences and Bioinformatics, School of Science, Xi'an Jiaotong-Liverpool University, Suzhou, Jiangsu, China

## Abstract

Sexual minoritized men (SMM) experience disproportionate rates of eating and body image disturbances relative to their heterosexual counterparts due, in part, to exposure to sociocultural pressures (e.g., community-specific body ideals, minority stress). Research often represents SMM as a monolith, precluding an understanding of within-group heterogeneity. There is a lack of research examining differences between SMM based on intimate partner preferences or sexual self-labels, including “top,” “bottom,” and “versatile.” The present study examined how thinness- and muscularity-oriented eating and body image disturbances and sociocultural variables (i.e., sexual minority and intracommunity stressors, tripartite influence model variables) differ across sexual self-label subgroups, including those not identifying with any label. Participants were SMM between the ages of 18 and 30 (*N*=375; tops, *n*=104, bottoms, *n*=60, versatiles, *n*=175, no self-label, *n*=36) recruited via Prolific. Results indicated significant group differences in muscularity-oriented eating and body image disturbances, thin internalization, internalized heterosexism, and body stigma intracommunity stress. Versatiles reported greater muscularity-oriented disordered eating than non-self-labels. Bottoms reported greater muscularity-oriented body dissatisfaction than tops, versatiles, and non-self-labels. Bottoms also reported greater thin internalization than tops. Individuals not identifying with any sexual self-label reported greater internalized heterosexism than versatiles and greater body stigma intracommunity stress than all other groups. Findings highlight the role of sexual self-labels in explaining meaningful heterogeneity in eating and body image disturbances and sociocultural stressors among SMM. Future research should replicate and extend our analyses to elucidate the temporal pathways underlying these associations.

## Introduction

### Eating and Body Image Disturbances in Sexual Minoritized Men

Relative to their heterosexual counterparts, sexual minoritized men (SMM; gay, bisexual, and other men who have sex with men) experience disproportionate rates of eating and body image disturbances related to both thinness and muscularity (Grunewald et al., 2021; Nowicki et al., 2022). While thinness-oriented concerns center around reducing body fat (via behaviors such as dietary restriction and excessive exercise) in pursuit of thin ideals, muscularity-oriented concerns involve preoccupation with increasing muscle size and definition (via behaviors such as anabolic steroid use and excessive protein consumption) in pursuit of muscular ideals (Calzo et al., 2017; Lev Arey et al., 2025). SMM’s vulnerability to these eating and body image disturbances may be linked to the unique appearance standards that exist within their community, such as pressure to achieve the ‘mesomorphic’ body-ideal, which emphasizes both a low percentage of body fat *and* a high degree of muscular hypertrophy (Convertino et al., 2022). Additionally, minority-specific stressors, including the marginalization of non-heterosexual orientations, adversely impact psychological health among SMM, with implications for eating and body image disturbances (Barnhart et al., 2022; Convertino et al., 2021b). Importantly, these eating and body image outcomes, as well as the associated sociocultural stressors, may differ across distinct *subgroups* of SMM, yet much of the existing research in this area has represented this population as a monolith. Emerging research identifies the role of sexual self-labels in describing the heterogeneous experiences of Chinese SMM, including those related to eating and body image disturbances (Barnhart et al., 2024).

### Understanding Sexual Self-Labels

Sexual self-labels refer to sexual behaviors and intimate partner preferences among SMM, including “top,” “bottom,” or “versatile.” These labels denote preferences for insertive (i.e., “top”) or receptive (i.e., “bottom”) intercourse, or those who adopt either position in a given occasion of intercourse (i.e., “versatile”), respectively (Hart et al., 2003; Sanderson, 1994). It is also possible, however, for an individual to not identify with any of these categories, which may carry important implications. For example, a lack of adherence to sexual self-labels may suggest low acculturation with the SMM community (Hart et al., 2003). This may lend itself to rejection from the community, underscoring negative psychosocial outcomes. Alternatively, low acculturation (i.e., not identifying with a sexual self-label) may also protect against such outcomes, as prior research has linked LGBTQ+ community involvement (i.e., participation in the mainstream gay community) to within-community stress experiences and eating and body image disturbances (Barnhart et al., 2026a, 2026b). Thus, there is a need to explore differences in those who identify with established sexual self-labels (top, bottom, and versatile) and those who do not identify with any of these labels in terms of sociocultural experiences and psychological outcomes, including eating and body image disturbances. An understanding of this kind may help inform theoretical models that contextualize the experiences of SMM by appreciating the unique, within-group heterogeneity of this vulnerable population, thus guiding hypotheses regarding identity-specific risk and protective factors as well as individualized mental health interventions.

### Psychosocial Correlates of Sexual Self-Labels

The process of constructing and recognizing sexual self-labels is complex and has also been shown to vary across cultures (Grov et al., 2018; Hart et al., 2003; Moskowitz & Roloff, 2017; Zeng et al., 2016). Additionally, these self-labels have been consistently associated with heteronormative gendered traits, whereby tops demonstrate profiles that are related to traditional standards of masculinity, such as aggressivity, strength, and dominance (Connell, 2005; Ravenhill & de Visser, 2018) compared to bottoms, who have often been stereotyped as possessing passive and effeminate qualities (Tskhay & Rule, 2013; Zheng et al., 2012) and may desire a partner with ‘gender typical’ (i.e., masculine) qualities (Moskowitz et al., 2008). On the other hand, those who identify as “versatile” may occupy a more fluid space that transcends gender-role stereotypes and allows for a balance of power-sharing in sexual relations (Kippax & Smith, 2001; Ravenhill & de Visser, 2018).

Importantly, these gendered personality traits and social roles are also closely associated with culturally prescribed body ideals, namely those which emphasize attributes historically conceptualized as masculine (i.e., strength, muscularity) or feminine (i.e., leanness) (Harris & Griffiths, 2023; Murnen & Don, 2012). The pursuit of these body ideals is associated with body dissatisfaction (when one perceives their appearance as discrepant from desired images) and, subsequently, disordered eating, to achieve socially prescribed muscularity- and/or thinness-oriented body ideals (Heider et al., 2018; Lavender et al., 2017). Considering that SMM are at increased risk for eating and body image disturbances and that this population is exposed to dual body ideals which emphasize both thinness *and* muscularity (Convertino et al., 2022), it is important to consider how sexual self-labels and their associated gendered traits converge with these body ideals to create heterogeneity in SMM’s experiences.

To date, only one study (Barnhart et al., 2024) has explored the overlap between sexual self-labels and eating and body image disturbances among SMM. This study examined differences in thinness- and muscularity-oriented concerns and psychosocial impairment among a sample of 403 SMM in China who reported top, bottom, and versatile sexual self-labels. Results indicated higher rates of thinness-oriented concerns among bottoms (e.g., body fat dissatisfaction; weight bias internalization) compared to tops and versatiles, and higher rates of muscularity-oriented concerns among tops, including muscularity internalization, drive for muscularity, and muscularity-oriented disordered eating. Interestingly, versatiles’ scores on eating and body image outcomes and psychosocial impairment were between those of their top and bottom counterparts, which is in line with the notion that versatiles may not solely ascribe to either masculine or feminine pressures and body ideals (Zheng & Fu, 2024). Taken together, these findings provide support for meaningful relationships between sexual self-label subgroups and eating and body image outcomes in the Chinese cultural context. However, this study did not account for individuals who do not identify with previously defined sexual self-labels, which represents an important gap in the literature, as there is potential for this subgroup to be distinct from those identifying as top, bottom, or versatile. Additionally, subgroup findings may differ between Eastern and Western populations (i.e., China and the United States, respectively) based on cultural norms, given that the Chinese context is historically marked by repressive attitudes towards sexual expression and dominated by heteronormative beliefs that may confer additional risk for SMM above and beyond that of their Western counterparts (Chen, 2020; Liu et al., 2011; Wang et al., 2019). Finally, to provide a more complete picture of the relationships between sexual self-labels and eating and body image outcomes, it is important to extend this research to include variables that represent foundational sociocultural frameworks—i.e., sexual minority and intracommunity stress, tripartite influence model—that are involved in the internalization of thinness- and muscularity-oriented body image ideals (Barnhart et al., 2024) and the experience of eating and body image disturbances more broadly in SMM (Convertino et al., 2021b; Pachankis et al., 2020; Soulliard et al., 2025).

### Sociocultural and Identity-Specific Stress as Frameworks to Understand Psychosocial Correlates of Sexual Self-Labels

Identity-specific stress models (i.e., sexual minority and intracommunity stress) represent key theoretical backdrops for understanding how and why eating and body image disturbances may manifest among SMM, and SMM of different sexual self-label groups may experience these stressors differently. According to minority stress theory (Brooks, 1981; Meyer, 2003) and intraminority gay community stress theory (Pachankis et al., 2020), SMM face excess exposure to distal and proximal stress, due to their stigmatized status as well as unique contextual pressures present within their immediate community, which are linked to adverse psychological and physical health sequelae, including eating and body image disturbances (Barnhart et al., 2022, 2026a, 2026b; Denning et al., 2025; Soulliard et al., 2024). Distal stressors characterize experiences external to an individual, including heterosexist discrimination, general intracommunity stress, and body stigma intracommunity stress. These stressors capture broad forms of discrimination and unique intracommunity pressures that emerge within an environment where social and sexual capital is derived, in part, from levels of physical attractiveness, desirability, and acceptability (Pachankis et al., 2020). Along these lines, the SMM community is marked by a focus on sex, status-based pressures that engender competition, and exclusion of diversity, all of which underscore intraminority stress processes (Pachankis et al., 2020). Proximal stressors, on the other hand, are internal to one’s experience, including internalized heterosexism and sexual orientation concealment, whereby one adopts negative stereotypes about their group and attempts to hide their identity to protect against these stereotypes, respectively (Frost & Meyer, 2023; Meyer, 2003).

Sexual self-label subgroups among SMM may experience sexual identity-based minority stress and intracommunity stress differently. For example, bottoms may experience pressure to conform to heteronormative gender experiences and ascribe to traditionally normed feminine characteristics associated with thin ideals (Barnhart et al., 2024). Alternatively, in line with femmephobia (i.e., the devaluation and denigration of femininity across gender and sexual identities; Hoskin, 2019, 2020), bottoms may encounter greater heterosexist discrimination related to self-ascribed effeminate traits, and this may confer pressure to ‘modify’ one’s body image and/or engage in identity concealment efforts to counteract negative stereotypes (Barnhart et al., 2024; Glick et al., 2007). Findings from qualitative research reflect these nuances, revealing narratives that capture the denigration of femininity among SMM and identify effeminate traits as a source of stigma within the community (Hammack et al., 2022). Versatiles may experience within-community rejection in the form of perceived illegitimacy of their self-label or related to sexual issues that could occur if a versatile partner desires to reverse their role, especially with a partner who is an intransigent top or bottom (Moskowitz et al., 2008). At the same time, the process of not ascribing to any sexual self-label may lend itself to increased intraminority stress, whereby one becomes more ‘distant’ from their community as a function of rejecting subcultural norms that are commonly used to facilitate social and sexual relations (i.e., sexual self-labels) among SMM. Alternatively, this same disconnection may also protect against pervasive pressures to achieve unattainable, socially-constructed appearance ideals, which may translate to reduced body stigma experiences from other SMM, which has implications for maladaptive eating behaviors in this population (Barnhart et al., 2026b).

Another model, which has previously been applied to understand experiences of SMM (Barnhart et al., 2022; Pasquariello et al., 2026), including their sociocultural and eating and body image experiences, is the tripartite influence model (TIM; Thompson et al., 1999; Tylka & Andorka, 2012). According to this model, pressure from social influences (i.e., peers, family, significant others, and the media) leads to the internalization (i.e., personal acceptance and self-directed beliefs about body ideals) of lean and muscular appearance ideals and appearance comparisons, whereby one evaluates their body against idealized standards. These processes, body-ideal internalizations and appearance comparisons, are demonstrated to underlie body dissatisfaction and subsequent disordered eating. The TIM has several strengths for understanding the experiences of SMM, one being its expansion to include ‘dual’ body image pathways, namely the involvement of both thinness- and muscularity-oriented processes. Further, recent research suggests that the integration of the TIM with minority stress theory explains meaningful variance in eating and body image outcomes, relative to either model alone (Barnhart et al., 2022; Convertino et al., 2021b; Frederick et al., 2022a, 2022b). Longitudinal research extends these findings, demonstrating significant prospective links between appearance pressures, appearance comparisons, and thinness- and muscularity-oriented eating and body image disturbances among SMM (Pasquariello et al., 2026).

However, by applying the processes implicated in the TIM to sexual self-label subgroups, there is an opportunity to clarify how exposure to and internalization of gendered appearance-based standards further contribute to heterogeneity in eating and body image disturbances among SMM. For example, tops may experience greater levels of muscularity-oriented concerns, in line with heteronormative appearance standards of masculinity and dominance, whereas bottoms may experience greater levels of thinness-oriented concerns, in line with standards of passivity and femininity (Barnhart et al., 2024; Zheng & Fu, 2024). Alternatively, those identifying as versatile may experience a ‘gradient’ of thinness- and muscularity-oriented concerns between those of their top and bottom counterparts (Barnhart et al., 2024). Those who do not identify with any sexual self-label may share vulnerabilities similar to SMM belonging to top, bottom, and/or versatile subgroups, and/or may be protected against certain elements of victimization (i.e., body stigma) by other SMM, though no research to date has explored these patterns.

### The Present Study

Responding to a dearth of empirical exploration in this area, the present study explores relationships between sexual self-labels, eating and body image disturbances, and sociocultural and identity-specific stressors among SMM. We extend previous research, which has begun to describe these associations in an Eastern context (i.e., China; Barnhart et al., 2024), by including a wider range of sociocultural processes and accounting for subgroups of SMM who do not identify with any sexual self-label. In integrating variables involved in minority stress frameworks and the TIM, including sexual minority and intracommunity stressors, sociocultural influences, and thinness- and muscularity-oriented eating and body image disturbances, findings advance our understanding of potential within-group heterogeneity among SMM, which may guide research directions in the service of informing prevention and intervention efforts for potential high-risk groups in this vulnerable population.

Given the paucity of scientific literature in this area, our study is largely exploratory in nature. However, there are several general patterns that we would expect to emerge with respect to eating and body image outcomes. In line with Barnhart et al.’s (2024) findings, we hypothesized that bottoms would report more thinness-oriented eating and body image disturbances, while tops would report more muscularity-oriented eating and body image disturbances, and versatiles would exhibit levels in between tops and bottoms. In line with the idea that sexual self-labels may reinforce eating and body image disturbances, we also expected that those who do not identify with any of these labels would exhibit lower levels of eating and body image disturbances overall. We did not formulate specific hypotheses regarding differences in sociocultural and minority stress outcomes across sexual self-label subgroups.

## Methods

### Participants and Procedure

Participants for the present study were recruited online via Prolific as part of a larger study examining eating behaviors, body image, and health in SMM. Recruitment occurred across two waves from January to September 2024 and November 2025 to June 2026 (ongoing). Eligibility criteria required that participants (a) were between 18 and 30 years old; (b) self-identified as gay, bisexual, or non-heterosexual; (c) self-identified as a cisgender man; and (d) were fluent in English and residing in the United States. The age range of 18–30 years was selected to be consistent with prior research in this population (Convertino et al., 2021b), given that young adults are at elevated risk for disordered eating.

The present study is a secondary data analysis of baseline data from a research project approved by the Institutional Review Board at Bowling Green State University (IRB # 2093126–5). All participants provided electronic informed consent prior to participation and were compensated $4.00 for providing valid data, consistent with Prolific payment policies and current minimum wage requirements in the United States. Valid data were defined as passing two out of three attention checks, completing responses throughout the survey, and correctly reporting on a qualitative entry item.

A total of *N* = 534 participants attempted to complete the baseline assessment (310 in the first wave, 224 in the second wave). Participants were excluded if they reported a transgender or gender diverse identity (*n* = 40), a heterosexual sexual orientation (*n* = 45), or an age greater than 30 years (*n* = 74). Baseline data for the remaining participant pool (*N* = 375) were included in the present study. On average, participants from the baseline data were bisexual (*n* = 229; 60.6%), white (*n* = 242; 64%), with “overweight” weight status (*M*_BMI_ = 28.71 [*SD* = 8.65]), and 25.43 years of age (*SD* = 3.01).

### Measures

#### Thinness-Oriented Outcomes

##### Body Dissatisfaction.

Thinness-oriented body dissatisfaction was assessed using the Body Dissatisfaction subscale of the Eating Disorder Inventory (EDI-BD; Garner et al., 1984). The EDI-BD is a 9-item measure assessing thinness-oriented body dissatisfaction (e.g., *“I think that my stomach is too big”*), rated on a 6-point Likert-type scale (1 = *Always*, 6 = *Never*), with five items reverse-scored. Items are summed to produce a total score, with higher scores indicating greater thinness-oriented body dissatisfaction. Previous research supports the internal consistency reliability of the EDI-BD in SMM (Barnhart et al., 2022; Barnhart et al., 2026b; Pasquariello et al., 2026). In this study, the internal consistency reliability of the measure was adequate (ω = 89).

##### Disordered Eating.

Thinness-oriented disordered eating was assessed using the Eating Disorder Examination-Questionnaire Short Form (EDE-QS; Gideon et al., 2016). The EDE-QS is a 12-item measure assessing thinness-oriented disordered eating over the past week (e.g., *“On how many of the past 7 days have you been deliberately trying to limit the amount of food you eat to influence your weight or shape (whether or not you have succeeded)?*”). Items 1–10 are rated on a 4-point response scale ranging from 1 = *0 days* to 4 = *6–7 days*, and items 11–12 are rated on a 4-point response scale ranging from 1 = *Not at all* to 4 = *Markedly*. A total score is calculated by summing all 12 items, with higher scores reflecting higher levels of thinness-oriented disordered eating. Previous research supports the internal consistency reliability of the EDE-QS in SMM (Barnhart et al., 2022). In this study, internal consistency reliability of the measure was adequate (ω = 91).

#### Muscularity-Oriented Outcomes

##### Body Dissatisfaction.

Muscularity-oriented body dissatisfaction was measured using the 7-item Attitudes Subscale of the Drive for Muscularity Scale (DMS; McCreary & Sasse, 2000). Participants responded to items on a 6-point Likert-type scale, with responses ranging from 1 (*Never*) to 6 (*Always*). In this study, the total score of the Attitudes Subscale was used, with higher scores indicating greater muscularity-oriented body dissatisfaction. In this study, the internal consistency reliability of the measure was adequate (ω = 91).

##### Disordered Eating.

Muscularity-oriented disordered eating was measured using the 15-item Muscularity-Oriented Eating Test (MOET; Murray et al., 2019). Participants rated items on a 5-point Likert-type scale, with responses ranging from 1 (*Never true*) to 5 (*Always true*). A total score was calculated, with higher scores indicating greater muscularity-oriented disordered eating. In this study, the internal consistency reliability of the measure was adequate (ω = 92).

#### Sexual Minoritized Stress

##### Heterosexist Discrimination.

Perceived exposure to sexual orientation-based discrimination over the past year was assessed using the 14-item Heterosexist Harassment, Rejection, and Discrimination Scale (HHRDS; Szymanski, 2009). This measure uses a 6-point scale ranging from 1 (*Neve*r) to 6 (*Almost all the time [more than 70% of the time]*). A total score was used to reflect overall levels of heterosexist discrimination, with higher scores reflecting a greater frequency of discrimination experiences. In this study, the HHRDS showed adequate internal consistency (ω = 93).

##### Internalized Heterosexism.

The 5-item Internalized Homophobia Scale-Revised (IHPR; Herek et al., 2009) was used to measure internalized heterosexism. Participants rated items on a 5-point Likert scale (1 = *Disagree strongly*; 5 = *Agree strongly*). An example item includes: “I wish I weren’t gay/bisexual.” A total score was used for this study, with higher scores indicating higher levels of internalized heterosexism. The internal consistency reliability of the IHP-R among SMM is supported by previous research (Herek et al., 2009). In the present study, McDonald’s omega of internalized heterosexism was adequate (ω = .90).

##### Sexual Orientation Concealment.

The 6-item Sexual Orientation Concealment Scale (SOCS; Jackson & Mohr, 2016) was used to assess the extent to which individuals conceal their sexual orientation. Participants rated items on a 5-point response scale (1 = *Not at all*; 5 = *All the time*) reflecting their experiences of sexual orientation concealment over the past two weeks. An example item includes: “In the last 2 weeks, I have remained silent while witnessing anti-gay remarks, jokes, or activities because I did not want to be labeled as LGB [Lesbian, Gay, Bisexual] by those involved.” We used a total score in this study, with higher scores indicating higher levels of sexual orientation concealment. Previous literature supports the internal consistency reliability of the SOCS among SMM (Jackson & Mohr, 2016). In the present study, McDonald’s omega of sexual orientation concealment was adequate (ω = .87).

#### Intracommunity Stress

##### Body Stigma Intraminority Stress.

The 3-item body stigma intraminority stress subscale was developed to assess body stigma specific to experiences of intraminority stress, or negative comments, discrimination, or judgment about one’s body from gay men in the community (Shepherd et al., 2023). Participants rated items on a 5-point Likert-type scale (1 = *Never*; 5 = *Very frequently*). An example item includes: “*I have been criticized for my body’s level of muscularity (too little or too much muscle)*.” We used a total score in this study, and higher scores indicate higher levels of body stigma intraminority stress. The internal consistency reliability of the body stigma intraminority stress subscale was supported among sexual minority men in the literature (Shepherd et al., 2023). In the present study, McDonald’s omega of body stigma intraminority stress scores was adequate (ω = .86).

##### General Intracommunity Stress.

The 8-item abbreviated Gay Community Stress Scale (GCSS; Maiolatesi et al., 2023; Pachankis et al., 2020) was used to assess general intraminority stress. The GCSS includes items assessing intraminority stressors in the domains of sex, social competition, status, and discrimination/exclusion of diversity. Participants rated items on a 5-point Likert-type scale (1 = *Not at all*; 5 = *Extremely*). An example item includes: “*The mainstream gay community values sex over meaningful relationships*.” We used subscale scores in this study that mapped onto distinct dimensions of general intracommunity stress, such as competition and jealousy among SMM, exclusion of diversity, pressures to engage in no-frills sex over meaningful relationships, and an emphasis on obtaining a high status (i.e., affluence). The internal consistency reliability of subscale scores of competition, exclusion, sex, and status on the GCSS was supported in SMM (Maiolatesi et al., 2023). Given each subscale consisted of 2 items, Spearman-Brown formula, using Pearson correlation coefficients, was applied: sex (.84), status (.86), competition (.84), and exclusion (.74).

#### Tripartite Influence Model

##### Appearance Pressures.

To assess appearance pressures, participants completed the pressures subscales of the Sociocultural Attitudes Toward Appearance Questionnaire 4-Revised (SATAQ-4R-Male; Schaefer et al., 2017). The 20 items included questions across four subscales, all rated on a 5-point Likert scale (1 = *Definitely disagree*, 5 = *Definitely agree*). Examples of items across the four subscales include: “I feel pressure from my family to look thinner” (family appearance pressures); “I feel pressure from my peers to look in better shape” (peers appearance pressures); “I feel pressure from my significant others to be more muscular” (significant others appearance pressures); and “I feel pressure from the media to decrease my level of body fat” (media appearance pressures). Aligning with previous research that emphasizes these four sources of pressure as distinct social agents (Schaefer et al., 2017), each subscale was totaled independently to reflect individual appearance pressure scores across family, peers, significant others, and the media, with higher scores indicating greater appearance pressures. Prior research using the gender-specific version of the SATAQ-4R among SMM has shown acceptable internal consistency reliability (Barnhart et al., 2022; Convertino et al., 2019). In the present study, internal consistency reliability was adequate across the family (ω = .86), peers (ω = .89), significant others (ω = .93), and media appearance pressures (ω = .93) subscales.

##### Thin and Muscular Body Ideal Internalization.

The SATAQ-4R-Male includes two subscales related to body-ideal internalization: thin and muscular body-ideal internalization. Participants responded to a total of 5 items (2 for the thin-ideal internalization subscale, 3 for the muscular-ideal internalization subscale) on a 5-point Likert scale (1 = *Definitely disagree*, 5 = *Definitely agree*). Example items are “I think a lot about looking thin” and “I want my body to look muscular” for thin and muscular body ideal internalization, respectively. Higher scores indicated greater levels of thin and muscular body-ideal internalization. Prior research has demonstrated the acceptability of the internal consistency reliabilities of the thin and muscular body-ideal internalization subscales in SMM (Barnhart et al., 2022; Convertino et al., 2019). In the current study, internal consistency was adequate for the muscular internalization subscale (ω = .93). Spearman-Brown formula, using Pearson correlation coefficients, was applied for thin internalization given it consisted of two items: .83.

##### Appearance Comparisons.

The 25-item frequency subscale of the Physical Appearance Comparison Scale-3 (PAC-3; Schaefer & Thompson, 2018) was used to assess appearance comparisons. Using a 5-point Likert-type scale (1 = *Never*, 5 = *Almost always*), participants rated the frequency with which they compare their appearance to others under certain circumstances (e.g., *“When I watch television, I compare my overall appearance to the appearance of the actors/actresses”*). An average score reflecting the overall level of appearance comparisons is calculated, with higher scores reflecting higher levels of appearance comparisons. Previous research supports the psychometric properties of the PAC-3 in men (Schafer & Thompson, 2018), and strong internal consistency reliability has been evidenced in SMM (Pasquariello et al., 2026). In the current study, internal consistency reliability was adequate for the frequency of appearance comparisons subscale (ω = .95).

### Analytical Plan

Analyses were conducted in SPSS v.30 (IBM Corp, 2024). A one-way analysis of variance (ANOVA) using *F* tests examined potential significant mean differences in variables among SMM reporting four sexual self-label categories: top, bottom, versatile, and no sexual self-label. Homogeneity of variances was explored. When equal variances could not be assumed, Welch’s *F* test was used to infer the presence of significant mean differences across sexual self-label groups. Visual inspection of Q-Q plots and histograms was conducted to assess the normality of residuals, which were confirmed with these data. With significant *F* tests, partial eta-squared values are generated, with .01, .06, and .14 indicating small, medium, and large effects, respectively (Cohen, 1992). Tukey post-hoc tests, which aid in controlling for Type 1 errors with multiple testing, were then examined to identify specific sexual self-label groups differences. As such, additional corrections for Type 1 error (e.g., Bonferroni) are not needed and were not applied in addition to Tukey post-hoc tests. With post-hoc tests, Cohen’s *d* values were used to indicate the effect size of significant mean differences between two sexual self-label groups (e.g., top vs. bottom). Cohen’s *d* effect sizes of 0.20, 0.50, and 0.80 indicate small, medium, and large effect sizes, respectively (Cohen, 1992). Finally, using G*Power 3.1 (Faul et al., 2009) to determine the minimum sample size to detect a medium effect size (Cohen’s *f* = 0.25; Cohen, 1992) with parameters set as significance < .05 and statistical power at 0.80, a sample size of 159 was identified. Thus, the present sample of 375 was adequate to detect large and medium effect sizes.

## Results

### Preliminary Analyses

To identify the presence of meaningful sociodemographic variables among SMM with different sexual self-labels, we conducted a one-way ANOVA examining group differences in age and BMI, both continuous variables. While the model examining age was not significant, the model examining BMI was significant (*F*(3, 368) = 2.75, *p* = .04); however, post-hoc results suggested no significant mean differences in BMI across sexual self-label groups. Furthermore, we examined the presence of differences in study variables with an analysis of covariance (ANCOVA) to determine if sexual orientation played a role, including SMM identifying as gay, bisexual, or other non-heterosexual sexual orientations (e.g., queer). Significant mean differences observed with the one-way ANOVA remained unchanged with the ANCOVA results, suggesting that sexual orientation did not play a role in the observed differences in eating and body image disturbances and sociocultural stressors.

### Descriptive and ANOVA Analyses

**Figure 1:**
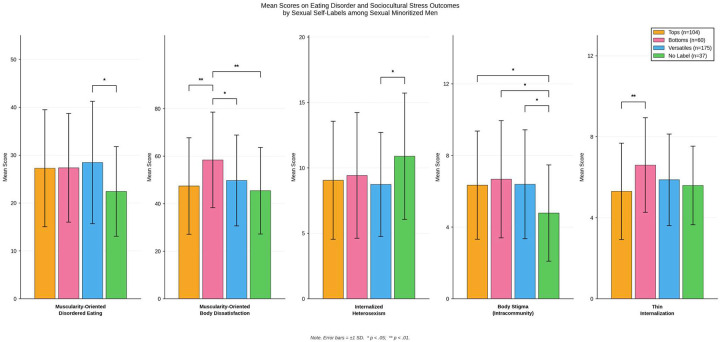
Mean scores and significant group differences

As shown in Table 1, of the 375 SMM, 104 (27.7%) identified as tops, 60 (16%) identified as bottoms, 175 (46.7%) identified as versatiles, and 36 (9.6%) did not identify with any sexual self-labels. Descriptive statistics of primary study variables by sexual self-labels are described in Figure 1 and Table 1.

Table 1 also shows the ANOVA, Tukey post-hoc results, and effect sizes. Regarding thinness- and muscularity-oriented eating and body image disturbances, the sexual self-label groups were significantly different in muscularity-oriented eating (Welch’s *F*(3, 129.76) = 3.71, *p* = .01, eta-squared = .02) and body image disturbances (*F*(3, 372) = 5.00, *p* = .002, eta-squared = .04). Because homogeneity of variances was not confirmed for muscularity-oriented disordered eating, Welch’s *F* test was reported for this model. Post-hoc (see Table 1) results showed that versatiles reported greater muscularity-oriented disordered eating than SMM with no sexual self-label (*d* = 0.54, *p* = .03, medium effect size). Bottoms reported greater muscularity-oriented body dissatisfaction than tops (*d* = 0.54, *p* = .003, medium effect size), versatiles (*d* = 0.44, *p* = .02, small effect size), and SMM with no sexual self-label (*d* = 0.68, *p* = .01, medium effect size).

Regarding sexual identity-based minority stress and intracommunity stress variables, the sexual self-label groups were significantly different in internalized heterosexism (*F*(3, 372) = 2.61, *p* = .050, eta-squared = .02), heterosexist discrimination (Welch’s *F*(3, 138.99) = 5.03, *p* = .002, eta-squared = .02), and body stigma intracommunity stress (*F*(3, 372) = 3.39, *p* = .02, eta-squared = .03). Because homogeneity of variances was not confirmed for heterosexist discrimination, Welch’s *F* test was reported for this model. Post-hoc results (see Table 1) revealed an approaching, but ultimately not significant difference between versatiles and SMM with no sexual self-label, suggesting versatiles reported more heterosexist discrimination than SMM with no sexual self-label (*d* = 0.53, *p* = .052, medium effect size). Interestingly, SMM with no sexual self-label reported greater internalized heterosexism than versatiles (*d* = 0.49, *p* = .03, small effect size). Finally, tops (*d* = 0.53, *p* = .04, medium effect size), bottoms (*d* = 0.63, *p* = .02, medium effect size), and versatiles (*d* = 0.56, *p* = .02, medium effect size) reported greater body stigma intracommunity stress than SMM with no sexual self-label.

Finally, regarding the tripartite influence model variables, the sexual self-label groups were significantly different in thin-ideal internalization (*F*(3, 372) = 4.301, *p* = .01, eta-squared = .03). Post-hoc results (see Table 1) showed that bottoms reported greater thin-ideal internalization than tops (*d* = 0.56, *p* = .003, medium effect size).

## Discussion

Sexual minoritized men (SMM) represent a heterogeneous population of individuals with diverse experiences; yet, the extant literature has relied on a monolithic perspective, which precludes understanding of how subgroups of SMM differ with respect to psychological outcomes, including eating and body image disturbances and sociocultural stressors. Sexual self-labels (i.e., sexual partner preferences) reflect such within-group variations and are associated with unique psychosocial correlates (i.e., eating and body image disturbances) among SMM (Barnhart et al., 2024; Zheng et al., 2012, 2015). There is a need to replicate and extend this research to explore how sexual self-label subgroups differ according to sociocultural stressors, and to consider the unique patterns that may emerge among those who do not identify with any sexual self-label. To this end, the present study examined associations between sexual self-labels (e.g., top, bottom, versatile, and no sexual self-label), eating and body image disturbances, and sociocultural stressors among SMM, providing support for the role of sexual self-labels in explaining meaningful heterogeneity in SMM’s experiences.

Regarding eating and body image disturbances, tops, bottoms, versatiles, and those who do not identify with any sexual self-label demonstrated unique patterns on some of these outcomes, namely, muscularity-oriented concerns. Versatiles reported significantly higher levels of muscularity-oriented disordered eating relative to SMM with no sexual self-label, and bottoms reported significantly higher levels of muscularity-oriented body dissatisfaction relative to their top, versatile, and no sexual self-label counterparts. These findings are somewhat counter to our hypotheses, in that, in line with previous research and gendered traits associated with masculinity and femininity (Kippax & Smith, 2001), we expected versatiles to fall along a gradient of eating and body image outcomes (Barnhart et al., 2024) and bottoms to demonstrate higher levels of thinness-oriented concerns. Instead, our finding that bottoms demonstrated higher levels of muscularity-oriented body dissatisfaction relative to all other sexual self-labels, including those who do not identify with these labels, carries several implications. For example, in line with femmephobia—i.e., negative attitudes towards femininity in men (Hoskin, 2019)—bottoms, who are perceived to possess more feminine attributes (i.e., submissive, small, slender; Johns et al., 2012) may experience a heightened awareness of these attributes, thus underlying elevated discontent with musculature and/or striving to appear more masculine to other SMM (Sánchez & Vilain, 2012). These strivings may be oriented towards accumulating erotic capital (i.e., quality and quantity of sexually desirable attributes; Smith et al., 2018) in the SMM community, of which bottoms may be afforded less compared to tops and versatiles (Wade et al., 2025) in an environment where elements of status are often closely tied to masculine gender norms (Shepherd et al., 2023). At the same time, as demonstrated by our sociocultural findings, namely those testing sexual self-label differences with key constructs implicated in the tripartite influence model, bottoms reported significantly higher levels of thin-ideal internalization relative to tops.

Taken together, these findings suggest that bottoms may be especially susceptible to the dual, unique eating and body image standards of SMM (Barnhart et al., 2024), which concurrently emphasize the pursuit of both thin *and* muscular ideals (Convertino et al., 2022; Tylka & Andorka, 2012). According to our models, these processes may exhibit distinct patterns among bottoms, whereby self-directed cognitions and internalized beliefs represent thinness-oriented concerns, while muscularity-oriented concerns manifest as attitudes towards one’s body image, and the extent to which one perceives their body as discrepant from societal ideals. This incorporation of thin and muscular body image concerns overlaps with mesomorphic body ideals (e.g., Calzo et al., 2017; Convertino et al., 2022) identified in SMM and contributes to recent research identifying that siloed thin and muscular body ideals may not represent body ideals in SMM (Pasquariello et al., 2026).

We also found that versatiles reported significantly higher levels of maladaptive eating behaviors, namely muscularity-oriented disordered eating, compared to those not identifying with any sexual self-label. It may be that, by not identifying with any sexual self-label, SMM are less likely to experience appearance-based pressures that are intertwined with intercourse preferences, including heteronormative body ideals, which may otherwise compound consequences related to eating and body image outcomes (Barnhart et al., 2024). On the other hand, versatiles’ report of greater muscularity-oriented disordered eating contrasts prior speculation that this subgroup may be more ‘freed’ from appearance-based pressures (Barnhart et al., 2024), to suggest that indeed, versatiles do experience these concerns at similar levels as their top and bottom counterparts. It is important to recognize, however, that versatiles were overrepresented in our sample (i.e., 46.7%), which led to imbalanced cell sizes (see Strengths, Limitations, and Future Directions for a discussion on this constraint), whereas in Barnhart et al.’s (2024) study, versatiles were relatively underrepresented compared to tops and bottoms. Additionally, this prior study was conducted with a Chinese sample, and these contrasting findings may speak to sociocultural frameworks unique to Western and non-Western contexts. For example, individuals in China may experience more rigid gendered expectations as a function of increased exposure to acute minority stress relative to their U.S. counterparts (Barnhart et al., revise and resubmit), and tops and bottoms, relative to their versatile counterparts, may be more likely to absorb the impact of appearance-based pressures in the Chinese cultural context (Barnhart et al., 2024; Yu & Sui, 2022). Our study benefits from the inclusion of these sociocultural variables, namely sexual minority and intracommunity stressors and the tripartite influence model, in explaining the experiences of sexual self-label subgroups in SMM.

Regarding sexual minority and intracommunity stressors, sexual self-label subgroups differed with respect to internalized heterosexism and body stigma intracommunity stress. We found that SMM not identifying with any sexual self-label reported significantly greater internalized heterosexism than versatiles, and that tops, bottoms, and versatiles reported greater body stigma intracommunity stress than SMM with no sexual self-label. Internalized heterosexism, which has been previously linked to eating and body image disturbances among SMM (Convertino et al., 2021a), refers to the process by which one “accepts,” or adopts societal prejudices and negative stereotypes about their sexual orientation (Meyer, 2003). Our findings on internalized heterosexism may suggest that not identifying with any sexual self-label lends itself to rejection of community, especially considering that self-labeling is a socially ‘normative’ process among SMM that conveys information relevant to partner choices and sociosexual hierarchies (Zheng et al., 2013). However, it is important to note that this difference emerged specifically relative to versatiles, suggesting that tops and bottoms shared similar levels of internalized heterosexism to no self-label SMM. Thus, this finding may also speak to unique differences of versatiles, such that the ability to be flexible in sexual roles (which are inherently gendered) liberates SMM from “heteronormative boxes,” as has been described in qualitative literature (Vytniorgu, 2025), in turn protecting against chronic internalization of heterosexist attitudes. Alternatively, as has been previously described (Zheng & Fu, 2024), it may be that SMM with internalized gender beliefs are more likely to have fixed self-labeling (i.e., exclusively tops or bottoms), while those reporting lower levels of internalized heterosexism feel less pressure to conform to gender stereotypical beliefs and are more likely to adopt flexible sexual self-labeling. Pending longitudinal models, these mechanistic and potentially bidirectional hypotheses are speculative in nature.

Finally, our findings on intracommunity body stigma suggest that not associating with sexual self-labels may buffer against community-specific pressures that reinforce body ideals and disordered eating in SMM (Barnhart et al., 2026b). This may be a function of no sexual self-label subgroups having reduced contact with other SMM (both platonic and romantic), which could diminish engagement with appearance comparisons and experiences of discriminatory pressures based on body size and shape. Our study did not measure community engagement in these interpersonal domains and in SMM specifically, though this points to an interesting future research direction.

Taken together, our findings underscore meaningful differences across sexual self-label subgroups, including those who do not identify with these labels, with respect to eating and body image outcomes and sociocultural stressors. Findings also speak to the complex nature of these associations, suggesting that, among SMM, sexual self-labeling—including not self-labeling—may buffer against or exacerbate certain eating and body image outcomes, while also reflecting level of acculturation to the community, carrying potential implications for expressions of sociocultural stress.

### Strengths, Limitations, and Future Directions

This study has several important strengths, as well as limitations that may guide future directions. To date, only one study has tested the relationships between sexual self-label subgroups and eating and body image disturbances in SMM (Barnhart et al., 2024); however, this study did not explore the implications of not identifying with any sexual self-label, nor did it apply minority stress and intraminority stress theories to understanding heterogeneity in SMM’s experiences. Thus, our study’s inclusion of a fourth group, namely those who do not adopt any sexual self-label, extends prior research in this area and identifies unique considerations regarding how this subgroup, as well as their sexual self-label counterparts, may be both protected against *and* more vulnerable to eating and body image outcomes. It is worth noting, however, that individuals in the no self-label subgroup comprised a relatively small percent of our sample (9.6%), and the distribution of tops, bottoms, and versatiles across our sample was relatively imbalanced—future research should replicate our findings in larger study populations and do so with evenly distributed sexual self-label subgroups. Additionally, to fully flesh out these nuances, longitudinal applications are needed to speak to the prospective associations between sexual self-labels, eating and body image disturbances, and sociocultural variables. For example, future research may consider exploring the role of sociocultural stressors (i.e., sexual identity-based minority stress and intracommunity stress) as prospective mediators in the pathways between sexual self-labels and eating and body image outcomes among SMM. Findings may lend understanding of the mechanisms that underlie discrepancies in eating and body image disturbances across sexual self-label subgroups over time, which would meaningfully contribute to theoretical models in this area and prevention and intervention efforts.

While our study benefits from the use of *Prolific*, which ensures high data validity, selfreport data is prone to recall bias (Rosenman et al., 2011). Thus, in addition to applying longitudinal methods, future research should consider approaches that incorporate time series data (i.e., ecological momentary assessment) to understand the dynamic interplay between eating and body image disturbances and sociocultural stress among SMM with different sexual self-label subgroups in real-time, in real-world environments. Additionally, our sample was non-clinical and sociodemographically constrained, which limits inferences regarding clinical populations (e.g., clinical eating disorders), as well as SMM belonging to diverse backgrounds. Future research should replicate our analyses to understand how clinical status and diverse identities may intersect—for example, sexual self-labels and racial/ethnic identity—to further classify the presence of meaningful subgroups according to SMM’s experiences.

### Conclusions

SMM face a range of sociocultural stressors that may increase the likelihood of thinness- and muscularity-oriented eating and body image disturbances (Barnhart et al., 2022, 2026a, 2026b, Shepherd et al., 2023; Soulliard et al., 2024). Responding to a body of literature that has overwhelmingly represented SMM as a monolith, the present findings suggest that meaningful heterogeneity across sociocultural stressors and eating and body image disturbances may be described, in part, by sexual self-labels. Compared to their top, versatile, and no self-label counterparts, bottoms demonstrated greater eating and body image disturbances relevant to both thin *and* muscular ideals. While versatiles demonstrated greater muscularity-oriented disordered eating relative to no self-labels SMM, no self-labels SMM demonstrated greater internalized heterosexism than versatiles. Further, tops, bottoms, and versatiles demonstrated more frequent experiences of intracommunity body stigma relative to no self-labels SMM. These findings suggest that sexual self-labeling represents a unique process that ties SMM to their community and may buffer against the incorporation of heterosexist attitudes into one’s self-concept, while also potentially increasing opportunities for exposure to body-image specific pressures from other SMM. Future research should continue to examine these subgroup differences and the temporal ordering of eating and body image and sociocultural processes, an understanding of which will be critical for the refinement of theoretical models and the development of prevention and intervention efforts for SMM.

## Figures and Tables

**Table 1:** Descriptive statistics and ANOVA results by sexual self-labels

Variable	Tops (*n* = 104)	Bottoms (*n* = 60)	Versatiles (*n* = 175)	No label (*n* = 37)	F	η^2^	Post hoc
	M (SD)	M (SD)	M (SD)	M (SD)			
TODE	21.75 (8.11)	22.08 (8.03)	22.03 (7.70)	19.89 (7.28)	0.80	.006	—
TOBD	28.98 (10.07)	26.75 (7.41)	28.49 (9.99)	26.70 (10.40)	1.02	.008	—
MODE^1^	27.25 (12.22)	27.35 (11.37)	28.46 (12.77)	22.41 (9.38)	3.74*	.020	Versatiles > No label
MOBD	47.41 (20.30)	58.40 (20.09)	49.74 (19.10)	45.43 (18.22)	5.00**	.039	Bottoms > Tops; Bottoms > Versatiles; Bottoms > No label
FAP	10.64 (4.69)	11.15 (5.42)	11.85 (5.34)	11.57 (5.12)	1.24	.010	—
PAP	10.12 (4.50)	9.25 (4.59)	10.35 (4.34)	9.76 (3.93)	1.00	.008	—
SOAP	11.23 (5.72)	10.75 (5.66)	11.74 (5.70)	10.16 (5.43)	1.03	.008	—
MAP	19.09 (7.84)	18.98 (8.12)	20.03 (7.06)	20.35 (7.58)	0.61	.005	—
MI	12.96 (4.27)	12.13 (4.66)	12.95 (4.25)	12.89 (3.72)	0.62	.005	—
TI	5.30 (2.38)	6.60 (2.34)	5.87 (2.26)	5.59 (1.94)	4.30**	.034	Bottoms > Tops
AC	22.09 (9.05)	23.13 (6.97)	23.02 (9.24)	22.19 (7.62)	0.34	.003	—
IH	9.05 (4.51)	9.42 (4.80)	8.73 (3.97)	10.89 (4.83)	2.61	.021	No label > Versatiles
HD^1^	22.43 (9.07)	24.63 (10.99)	25.03 (11.50)	20.22 (5.97)	5.03**	.024	—
SOC	11.72 (5.61)	11.88 (6.65)	12.00 (6.08)	11.62 (5.88)	0.07	.001	—
BSIS	6.34 (3.02)	6.67 (3.27)	6.39 (3.04)	4.78 (2.69)	3.39*	.027	Bottoms > No label; Tops > No label; Versatiles > No label
Status IS	2.81 (1.20)	2.99 (1.36)	2.82 (1.22)	2.58 (1.12)	0.85	.007	—
EIS	2.90 (1.12)	3.02 (1.26)	2.76 (1.16)	2.39 (1.16)	2.57	.020	—
Sex IS	3.27 (1.20)	3.58 (1.28)	3.12 (1.23)	3.04 (1.32)	2.45	.019	—
CIS	3.43 (1.11)	3.47 (1.24)	3.21 (1.19)	3.01 (1.31)	1.85	.015	—

Note.

* *p* < .05,

** *p* < .01,

TODE = thinness-oriented disordered eating, TOBD = thinness-oriented body dissatisfaction, MODE = muscularity-oriented disordered eating, MOBD = muscularity-oriented body dissatisfaction, FAP = family appearance pressures, PAP = peers appearance pressures, SOAP = significant others appearance pressures, MAP = media appearance pressures, MI = muscular internalization, TI = thin internalization, AC = appearance comparisons, IH = internalized heterosexism, HD = heterosexist discrimination, SOC = sexual orientation concealment, BSIS = body stigma intraminority stress, IS = intraminority stress, EIS = exclusion intraminority stress, CIS = competition intraminority stress.

1= Welch’s *F* statistic reported.

## Data Availability

These data are available from the corresponding author upon reasonable request.
